# Remnant lipoprotein cholesterol is associated with incident new onset diabetes after transplantation (NODAT) in renal transplant recipients: results of the TransplantLines Biobank and cohort Studies

**DOI:** 10.1186/s12933-022-01475-y

**Published:** 2022-03-16

**Authors:** Tamas Szili-Torok, Sara Sokooti, Maryse C. J. Osté, Antonio W. Gomes-Neto, Robin P. F. Dullaart, Stephan J. L. Bakker, Uwe J. F. Tietge

**Affiliations:** 1 Department of Internal Medicine, University Medical Center Groningen, University of Groningen, Groningen, The Netherlands; 2 Division of Clinical Chemistry, Department of Laboratory Medicine (LABMED), H5, Alfred Nobels Alle 8, Karolinska Institutet, 141 83 Stockholm, Sweden; 3 Clinical Chemistry, Karolinska University Laboratory, Karolinska University Hospital, Stockholm, Sweden

**Keywords:** Transplantation, Kidney, Diabetes, Cholesterol, Remnants, Prospective, Incident, Renal transplant recipients, NODAT, Complications

## Abstract

**Background:**

New onset diabetes after transplantation (NODAT) is a frequent and serious complication of renal transplantation resulting in worse graft and patient outcomes. The pathophysiology of NODAT is incompletely understood, and no prospective biomarkers have been established to predict NODAT risk in renal transplant recipients (RTR). The present work aimed to determine whether remnant lipoprotein (RLP) cholesterol could serve as such a biomarker that would also provide a novel target for therapeutic intervention.

**Methods:**

This longitudinal cohort study included 480 RTR free of diabetes at baseline. 53 patients (11%) were diagnosed with NODAT during a median [interquartile range, IQR] follow-up of 5.2 [4.1–5.8] years. RLP cholesterol was calculated by subtracting HDL and LDL cholesterol from total cholesterol values (all directly measured).

**Results:**

Baseline remnant cholesterol values were significantly higher in RTR who subsequently developed NODAT (0.9 [0.5–1.2] mmol/L vs. 0.6 [0.4–0.9] mmol/L, p = 0.001). Kaplan-Meier analysis showed that higher RLP cholesterol values were associated with an increased risk of incident NODAT (log rank test, p < 0.001). Cox regression demonstrated a significant longitudinal association between baseline RLP cholesterol levels and NODAT (HR, 2.27 [1.64–3.14] per 1 SD increase, p < 0.001) that remained after adjusting for plasma glucose and HbA1c (p = 0.002), HDL and LDL cholesterol (p = 0.008) and use of immunosuppressive medication (p < 0.001), among others. Adding baseline remnant cholesterol to the Framingham Diabetes Risk Score significantly improved NODAT prediction (change in C-statistic, p = 0.01).

**Conclusions:**

This study demonstrates that baseline RLP cholesterol levels strongly associate with incident NODAT independent of several other recognized risk factors.

## Background

Renal transplantation represents the preferred treatment option for patients with end-stage renal disease in terms of cost efficiency, gain in quality of life and decrease in morbidity and mortality [1]. With constantly increasing survival rates of renal transplant recipients (RTR) and improved immunosuppressive regimens, chronic complications of the stable long-term course after kidney transplantation become clinically more and more important [2, 3]. New Onset Diabetes After Transplantation (NODAT) is one such relevant condition which might affect up to 50% of RTR [4]. NODAT is associated with worse patient and graft outcomes mirrored by elevated risks of cardiovascular events and chronic graft failure [2, 4]. NODAT also significantly increases the yearly costs of post transplantation care estimated at $12,000 in the first year after transplantation and $19,000 during following years in the United States [5]. Despite the clinical importance of the topic, pathophysiological mechanisms favoring the development of NODAT are still ill explored and consequently current attempts to define predictive biomarkers have been of limited practical relevance [1, 2, 4]. A common denominator on which the pathophysiology of NODAT and type 2 diabetes converges appears to be proper functioning of beta cells [4]. Beta cells, however, are sensitive to disturbances in their cholesterol balance. Cholesterol loading of beta-cells via uptake of LDL renders them dysfunctional and impairs insulin secretion [6]. These observations can provide an explanation why the use of statins, that increase LDL receptor expression on beta-cells, is associated with an elevated risk of type 2 diabetes [7]. Indeed, we have recently shown that also in RTR statin use increases the risk of developing NODAT [8]. On the other hand, impaired HDL-mediated cholesterol efflux, a mechanism to unload cellular cholesterol, also seems to result in dysfunctional beta-cells [9]. Consistent with these data, we reported in recent observational work that a low baseline HDL-mediated cholesterol efflux capacity substantially increases the risk of incident NODAT in RTR during follow-up [10]. Apart from LDL and HDL, remnant lipoprotein (RLP) cholesterol represents an emerging subclass that prospectively associates not only with higher incident cardiovascular disease, but also with increased overall mortality and systemic inflammation [11, 12]. Remnants are incompletely lipolyzed remainders of liver-derived VLDLs and intestine-derived chylomicrons [11]. Conceivably, remnants could be even more damaging to beta-cells than LDL, since they are taken up in an unregulated fashion via scavenger receptors [11, 13]. However, to date no data exist clinically exploring such a hypothesis. Therefore, the current study was designed to test whether in RTR baseline levels of RLP cholesterol associate with the risk of incident NODAT.

## Methods

### Study design and study population

The research question was addressed in a prospective longitudinal cohort study from the Northern Netherlands [14]. Data collection and inclusion took place between November 2008 and June 2011 using the Transplantlines Food and Nutrition Biobank and Cohort study (NCT02811835). All RTR above the age of 18 with at least one year of functioning allograft visiting the outpatient clinic of the University Medical Center Groningen were eligible to join. Exclusion criteria comprised congestive heart failure, cancer other than cured skin cancer and endocrine disorders other than diabetes mellitus. Out of 817 eligible patients 707 (86.5%) gave written informed consent. Participants did not differ in baseline characteristics from all eligible patients. Patients with missing values of either remnant cholesterol or to establish a diagnosis of NODAT (n = 57) or with a history of diabetes or glucose lowering medication use (n = 169) were excluded from the study, leaving 480 RTR eligible for the current analysis.

This study was approved by the local Institutional Review Board (METc 2008/186) and was conducted according to the Declaration of Helsinki.

### Outcome measures and end point

The primary outcome measure was fasting plasma RLP cholesterol.

The primary end point was incident NODAT during follow-up. NODAT was defined in accordance with the latest Expert Panel recommendations from the American Diabetes Association criteria with the following requirements [15]: fasting plasma glucose concentration > 126 mg/dL (7.0 mmol/L); non-fasting plasma glucose concentration > 200 mg/dL (11 mmol/L), HbA1c level ≥ 6.5% (53 mmol/mol); use of glucose lowering drugs and/or typical symptoms (polyuria, polydipsia, unexplained weight loss).

### Measurements and definitions

Information on medication and medical history were derived from patient records. BMI was calculated as weight (kg) divided by height squared (m^2^). Blood pressure was measured three times using an automated device (every minute for 15 min using Dinamap1846; Critikon, Tampa, FL) and the average of the three measurements was calculated. Waist circumference was measured on skin midway between the iliac crest and the 10^th^ rib.

All blood samples were drawn after an overnight fast of 8–12 h. Routine clinical chemistry methods were used to determine plasma total cholesterol (cholesterol oxidase-phenol aminophenazone method, MEGA AU 510; Merck Diagnostica, Darmstadt, Germany), HDL cholesterol (cholesterol oxidase-phenol aminophenazone method on a Technikon RA-1000, Bayer Diagnostics, Mijdrecht, The Netherlands) and triglyceride levels (glycerol-3-phosphate oxidase-phenol aminophenazone method, Roche Diagnostics, Basel, Switzerland). LDL cholesterol was measured directly using a Roche P-modular automated analyzer (Roche Diagnostics). This allowed for an accurate calculation of plasma remnant cholesterol concentrations by subtracting values of HDL cholesterol and LDL cholesterol from total cholesterol levels [16]. Plasma hsCRP was assessed using an immunoturbidimetric assay (Roche Modular P Chemistry platform, Roche Diagnostics, Manheim, Germany). Plasma urine and creatinine concentrations were determined using an isotope dilution mass spectrometry (IDMS) traceable enzymatic method on a Roche P-modular automated analyzer. Renal function was assessed using the combined creatinine cystatin C-based Chronic Kidney Disease Epidemiology Collaboration (CKD-EPI) formula in order to calculate the estimated glomerular filtration rate (eGFR). HbA1c concentrations were measured using a turbidimetric inhibition immunoassay (Roche Integra). Total urinary protein excretion was determined utilizing the Biuret reaction (MEGA AU 510, Merck Diagnostica). Proteinuria was defined as a protein excretion of ≥ 0.5 g per 24 h. Urinary albumin was determined using nephelometry (Dade Behring Diagnostics).

### Statistical analysis

A P value of < 0.05 was considered to be statistically significant. All statistical analyses were performed using the Statistical Package for the Social Sciences version 26 (IBM SPSS) and RStudio (RStudio Team, 2020, RStudio: Integrated Development for R. RStudio, PBC, Boston, MA). All variables were checked for normal distribution. Data with normal distribution are expressed as mean ± standard deviation (differences tested using one way ANOVA) and data with skewed distribution are expressed as median [interquartile range, IQR] (differences tested with the Kruskall-Wallis test). Absolute numbers (percentages) are given for categorical variables (p values for differences obtained by χ^2^-test). The development of NODAT was visualized using Kaplan–Meier analysis and statistical significance was tested utilizing the log rank (Mantel-Cox) test [17]. To inform about the functional association between RLP cholesterol and NODAT, cubic splines analysis was done with four knots. Cox regression analysis with the spline term was performed on 4 models: crude, adjusted for age and sex, adjusted for age, sex and HbA1c and adjusted for age, sex, HbA1c and BMI. The relative risk for NODAT at different RLP cholesterol concentrations was plotted. To adjust for relevant confounders Cox proportional hazards regression analysis was performed. The following seven models were constructed: model 1 (crude analysis), model 2 (adjusted for age and sex), model 3 (model 2 + BMI, systolic and diastolic blood pressure), model 4 (model 2 + eGFR, time since renal transplantation, acute rejection, HLA class I and II antibodies), model 5 (model 2 + urinary albumin to creatinine ratio (ACR), model 6 (model 2 + plasma glucose, HbA1c), model 7 (model 2 + HDL cholesterol, LDL cholesterol), model 8 (model 2 + statin use), model 9 (model 2 + smoking and alcohol consumption), model 10 (model 2 + use of proliferation inhibitors, calcineurin inhibitors, tacrolimus, cyclosporine and prednisolone dose). Using Schoenfeld residuals test the proportional hazard assumption was found not to be violated. Subsequently, sensitivity analyses with the same models were performed in RTR excluding those with impaired fasting glucose (IFG) (defined according to the WHO criteria as a fasting plasma glucose between 6.1 and 7.0 mmol/l). In addition, subgroup analyses using interaction tests were performed in which HR were determined across categories of baseline characteristics. For continuous variables the median value was used as cut-off. Included characteristics were sex, age, smoking, alcohol consumption, BMI, eGFR, cyclosporine and tacrolimus use as well as daily prednisolone dose. We calculated the Framingham Diabetes Risk Score in all participants (using the parameters age, sex, BMI, blood pressure, HDL cholesterol, triglycerides and fasting glucose). We did not include family history in the score, since this parameter is not strictly objective [18] and it was not consistently available in all participants. In order to test the predictive capacity of the risk score, forward stepwise conditional binary logistic regression was carried out and in the first step remnant cholesterol was added in order to estimate whether this increases the predictive capacity of the model. Furthermore, C-statistics was done for the Framingham Diabetes Risk Score in a crude Cox regression model. Subsequently, RLP-C was added to this model and the concordance was determined. P values representing the improvement in the models were determined using ANOVA.

## Results

The median age of the participants was 53.4 [42.8–62.1] years at baseline with 57.5% being male. When patients were divided into sex-stratified tertiles of RLP cholesterol the respective median values were 0.3 [0.2–0.4] for the low, 0.6 [0.5–0.7] for the medium and 1.1 [0.9–1.4] mmol/L for the high tertile (Table 1). Higher RLP cholesterol concentrations were significantly associated with (i) increased BMI, (ii) a worse allograft function as reflected by increased serum creatinine and a lower eGFR, and (iii) dyslipidemia, as expected, especially lower HDL cholesterol and higher total cholesterol, LDL cholesterol as well as triglyceride levels. Higher RLP cholesterol levels were not significantly associated with other constituents of the metabolic syndrome, such as higher waist circumference, higher systolic blood pressure, and higher glucose or with higher circulating hsCRP. There was also no statistically significant association detected between HbA1c and RLP cholesterol. With respect to medication, antihypertensives (p = 0.864), statins (p = 0.165), cyclosporine (p = 0.490), tacrolimus (p = 0.686) and prednisolone (p = 0.183) use were not different among the RLP cholesterol tertiles.

**Table 1:** Baseline characteristics according to sex-stratified tertiles of RLP cholesterol

Variable (n = 480)	T1 (low tertile), n = 151	T2 (middle tertile), n = 180	T3 (high tertile), n = 149	P value for trend
RLP cholesterol (mmol/L)	0.3 [0.2–0.4]	0.6 [0.5–0.7]	1.1 [0.9–1.4]	< 0.001
General characteristics				
Age (years)	53.6 [40.7–62.3]	53.8 [41.3–62.7]	53.1 [44.7–61.3]	0.902
Male sex (%)	49.0	63.3	59.1	0.029
Smoking status				
Never smoker (%)	47.9	40.8	37.0	0.159
Former smoker (%)	41.7	49.4	41.8	0.271
Current smoker (%)	10.4	9.8	21.2	0.005
Alcohol consumption				
None (%)	12.7	8.4	9.5	0.430
0–10 g/day (%)	63.3	59.6	59.9	0.750
10–30 g/day (%)	18.0	26.4	25.2	0.166
> 30 g/day (%)	6.0	5.6	5.4	0.978
Body composition				
BMI (kg/m^2^)	24.8 [22.1–28.1]	25.6 [23.2–27.7]	26.4 [23.3–29.9]	0.017
Waist circumference (cm)	95.0 ± 15.7	96.3 ± 13.5	98.6 ± 13.1	0.096
Transplant history				
Time since renal transplantation (years)	7.0 [4.0–12.0]	6.0 [2.3–12.0]	4.0 [1.0–12.0]	0.019
Deceased donor (%)	65.5	64.4	63.1	0.904
Donor age (years)	44.0 [29.0–53.0]	46.0 [31.0–55.8]	48.0 [34.0–56.0]	0.263
Dialysis duration (months)	42.5 [13.0–62.8]	48.0 [23.0–64.5]	32.0 [16.0–54.0]	0.234
Acute rejection (%)	19.2	25.0	27.5	0.221
HLA class I positive (%)	10.6	11.1	10.1	0.700
HLA class II positive (%)	14.6	11.7	10.7	0.819
Renal allograft function				
Serum creatinine (µmol/L)	112.0 [92.0–140.3]	122.0 [101.0–152.0]	139.0 [109.0–178.5]	< 0.001
eGFR (mL/min/1.73 m^2^)	55.9 [42.4–70.2]	52.9 [42.3–64.1]	43.9 [30.3–61.0]	< 0.001
Urinary albumin-to-creatinine ratio (UACR)	28.4 [6.6–100.5]	25.3 [7.8–115.2]	32.3 [7.8–158.6]	0.683
Proteinuria (≥ 0.5 g/24 h) (%)	17.2	22.2	21.5	0.493
Inflammation markers				
hsC-reactive protein (mg/L)	1.2 [0.5–3.3]	1.4 [0.6–4.5]	1.6 [0.8–4.0]	0.254
Blood pressure				
Diastolic blood pressure (mmHg)	82.9 ± 11.3	82.3 ± 10.9	83.9 ± 10.5	0.447
Systolic blood pressure (mmHg)	133.0 [122.0–142.0]	135.0 [125.0–146.0]	135.0 [125.0–145.5]	0.297
Glucose homeostasis				
Plasma glucose (mmol/L)	5.1 [4.7–5.4]	5.0 [4.7–5.5]	5.2 [4.7–5.6]	0.605
HbA1c (mmol/mol)	38.0 [36.0–41.0]	39.0 [36.0–41.0]	39 [36.0–42.0]	0.479
HbA1c (%)	5.6 [5.4–5.9]	5.7 [5.4–5.9]	5.7 [5.4–6.0]	0.479
Lipids and lipoproteins				
Total cholesterol (mmol/L)	4.7 [4.1–5.5]	4.8 [4.3–5.5]	5.6 [5.0–6.3]	< 0.001
LDL cholesterol (mmol/L)	2.8 [2.2–3.4]	2.8 [2.3–3.3]	3.0 [2.6–3.6]	0.004
HDL cholesterol (mmol/L)	1.5 [1.2–1.9]	1.3 [1.1–1.6]	1.2 [1.0–1.4]	< 0.001
Triglycerides (mmol/L)	1.2 [1.0–1.4]	1.6 [1.3–1.9]	2.4 [1.9–3.0]	< 0.001
Medication use				
Antihypertensives (%)	85.4	87.2	87.2	0.864
Statins (%)	43.7	51.1	54.4	0.165
Proliferation inhibitor (%)	84.1	86.7	83.9	0.730
Calcineurin inhibitor (%)	46.4	59.4	59.1	0.030
Tacrolimus (%)	13.2	15.6	16.8	0.686
Cyclosporine (%)	33.1	39.4	36.2	0.490
Prednisolone (mg/24 h)	10.0 [7.5–10.0]	10.0 [7.5–10.0]	10.0 [7.5–10.0]	0.183

Continuous data with normal distribution are shown as mean ± standard deviation, differences were tested using one-way ANOVA. Continuous data with skewed distribution are shown as median [IQR] and the differences were tested using Kruskal–Wallis test. Categorical data are shown as n (%) and differences were analyzed using the chi-square test

**Fig. 1:**
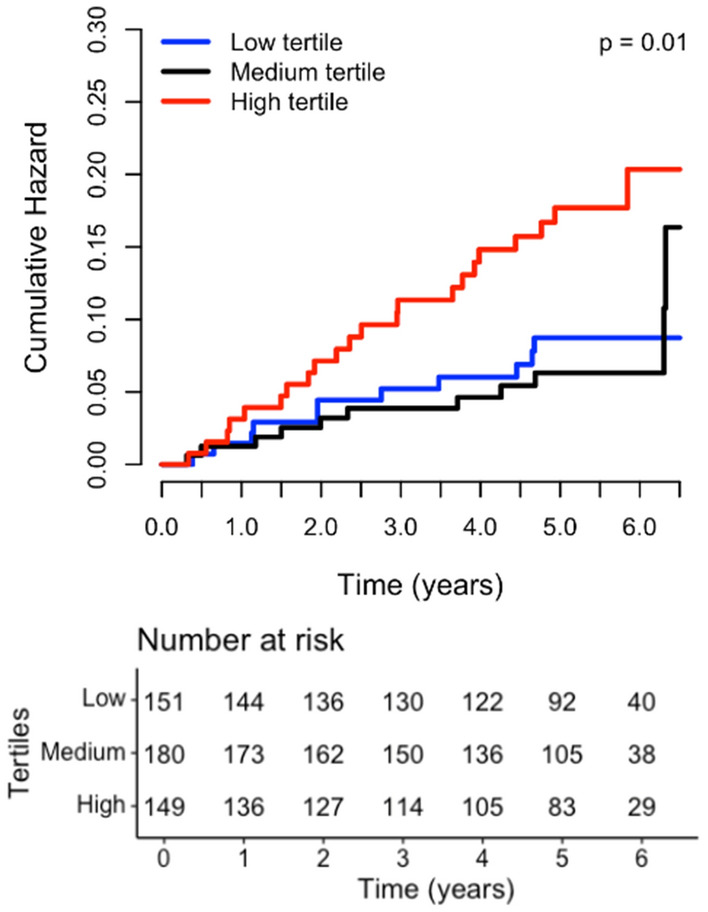
Kaplan–Meier analysis (log rank test: p = 0.01)

**Fig. 2:**
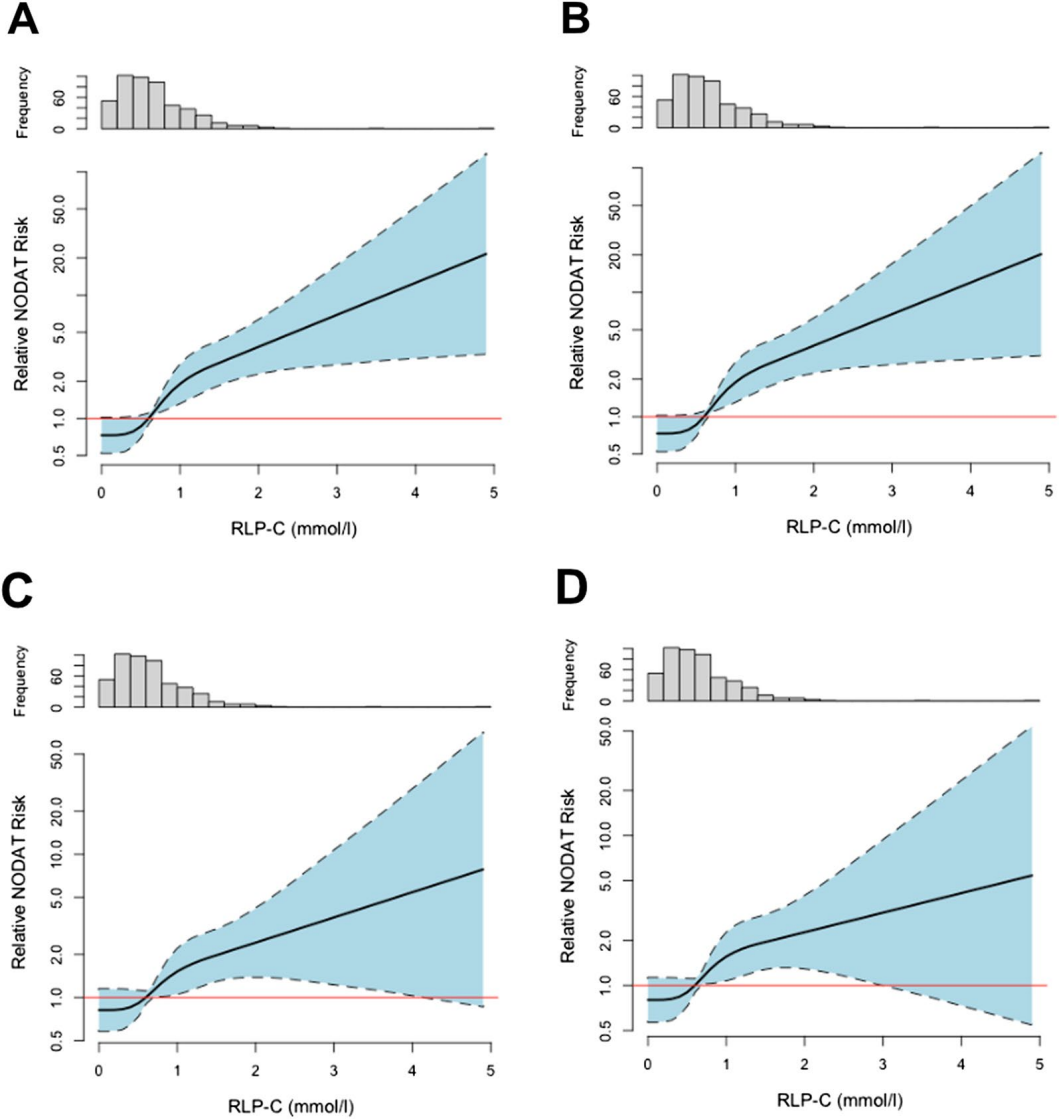
Probability of incident NODAT according to remnant lipoprotein cholesterol (RLP-C) levels. Probabilities were determined by Cox regression analysis using cubic splines with four knots. Please note the logarithmic scale of the y-axis. **A** crude analysis, **B** adjusted for age and sex, **C** adjusted for age, sex and HbA1c, **D** adjusted for age, sex, HbA1c and body mass index (BMI). The knots are located at 0.1, 0.5, 0.8 and 1.5 mmol/l

During a median follow-up of 5.2 [4.1–5.8] years, 53 patients developed NODAT (11%). Baseline remnant cholesterol values were significantly higher in those who subsequently developed NODAT (0.9 [0.5–1.2] mmol/L) vs. those who did not (0.6 [0.4–0.9] mmol/L, p = 0.001). To explore a longitudinal association between NODAT and plasma RLP cholesterol first Kaplan–Meier curves were plotted. A highly significant association between high RLP cholesterol and incident NODAT was seen (log rank test p = 0.01, Fig. 1). Next, Cox proportional hazard analyses were carried out (Table 2). In crude analysis (model 1), RLP cholesterol was prospectively associated with NODAT (HR, 2.27 [1.64–3.14] per 1 SD increase, p < 0.001), an association which remained materially unchanged when adjusting for age and sex (model 2, HR, 2.24 [1.62–3.11], p < 0.001). Adding BMI and blood pressure to model 2 (model 3) did also not substantially alter this association (HR, 1.81 [1.29–2.53], p < 0.001). Subsequently, also after adjustment for renal function, time since transplantation, acute rejection and HLA class I and II antibodies in model 4 (HR, 2.34 [1.63–3.36], p < 0.01), for urinary albumin-creatinine ratio (UACR) in model 5 (HR, 2.14 [1.52, 3.02], p < 0.001), for plasma glucose and HbA1c in model 6 (HR, 1.80 [1.23–2.64], p = 0.002) and for HDL and LDL cholesterol in model 7 (HR, 1.68 [1.15–2.47], p = 0.008) the prospective association between RLP cholesterol and incident NODAT remained significant. In addition, after adjusting for statin use in model 8 (HR, 2.23 [1.59–3.12], p < 0.001), smoking and alcohol consumption in model 9 (HR, 2.33 [1.61, 3.38], p < 0.001) and immunosuppressive medication use in model 10 (HR, 2.14 [1.51–3.05], p < 0.001) the conclusion reached from previous models did not change. Sensitivity analysis using the same models in RTR without IFG at baseline showed a comparable strong association between baseline RLP cholesterol and incident NODAT (Table 2). When dichotomizing RTR by several participant level characteristics the prospective association of RLP cholesterol with incident NODAT was not different for males vs. females (p value for interaction = 0.639), participants older or younger than 53.4 years (p = 0.498), smokers vs. non-smokers (p = 0.709), an alcohol consumption below or ≥ 10 mg/d (p = 0.434), a BMI < or ≥ 24.4 kg/m^2^ (p = 0.373), an eGFR < or ≥ 40.2 mL/min/1.73m^2^ (p = 0.725), a prednisolone dose < or ≥ 10 mg/d (p = 0.659) and for those using vs. not using cyclosporine (p = 0.118) or tacrolimus (p = 0.862). Restricted cubic spline analysis (Fig. 2) showed a continuous increase in relative NODAT risk with increasing RLP cholesterol levels both in crude as well as adjusted analyses.

**Table 2:** Association of 1 mmol/l increase in RLP cholesterol levels with incident NODAT as determined by Cox regression analysis

		All RTR (n = 480)		RTR with IFG excluded (n = 432)	
		HR [95% CI]	P value	HR [95% CI]	P value
Model 1	Crude analysis	2.27 [1.64–3.14]	< 0.001	2.21 [1.55, 3.17]	< 0.001
Model 2	Adjusted for age and sex	2.24 [1.62–3.11]	< 0.001	2.18 [1.52, 3.11]	< 0.001
Model 3	Model 2 + BMI, systolic and diastolic blood pressure	1.81 [1.29–2.53]	< 0.001	1.72 [1.18, 2.49]	0.004
Model 4	Model 2 + eGFR and time since transplantation, acute rejection, HLA class I and II antibodies	2.34 [1.63–3.36]	< 0.001	2.0 [1.36, 2.94]	< 0.001
Model 5	Model 2 + UACR	2.23 [1.61, 3.09]	< 0.001	2.17 [1.52, 3.1]	< 0.001
Model 6	Model 2 + plasma glucose, HbA1c	1.80 [1.23–2.64]	0.002	1.72 [1.14, 2.58]	0.009
Model 7	Model 2 + HDL cholesterol, LDL cholesterol	1.68 [1.15–2.47]	0.008	1.65 [1.09, 2.51]	0.019
Model 8	Model 2 + statin use	2.23 [1.59–3.12]	< 0.001	2.09 [1.45, 3.02]	< 0.001
Model 9	Model 2 + smoking and alcohol use	2.33 [1.61, 3.38]	< 0.001	2.2 [1.46, 3.3]	< 0.001
Model 10	Model 2 + use of proliferation inhibitors, calcineurin inhibitors, tacrolimus, cyclosporine and prednisolone dose	2.14 [1.51–3.05]	< 0.001	2.09 [1.42, 3.06]	< 0.001

*IFG* impaired fasting glucose, *BMI* body mass index, *eGFR* estimated glomerular filtration rate, *HLA* human leukocyte antigen, *UACR* urinary albumin-to-creatinine ratio, *HDL* high density lipoprotein, *LDL* low density lipoprotein

No validated generally accepted model for NODAT prediction has been established in RTR. The Framingham Diabetes Risk Score is frequently used [19]. The risk score was able to also predict incident NODAT in our cohort (OR [95% CI], 1.09 [1.04–1.14], p < 0.001, Table 3). Adding RLP cholesterol to the model (OR, 2.04 [1.18–3.53]. p = 0.011) resulted in a significant improvement in NODAT prediction (p = 0.009, Table 3). Determining the concordance scores for models including the Framingham Diabetes Risk Score with and without RLP cholesterol showed that the addition of RLP cholesterol significantly improved the fit of the model (p < 0.01, Table 3).

**Table 3:** Logistic regression analysis of the Framingham Diabetes Risk Score without and with the addition of RLP cholesterol

Stepwise logistic regression				
	Model	OR [95% CI]	P value	P value from previous step
Step 0	Framingham Diabetes Risk Score	1.09 [1.04–1.14]	< 0.001	
Step 1	Addition of RLP cholesterol	2.04 [1.18–3.53]	0.011	0.009

C-statistics				
		Concordance	∆Concordance	P value
Framingham Diabetes Risk Score		0.671		
Framingham Diabetes Risk score and RLP cholesterol		0.686	+ 0.015	0.01

## Discussion

The results of this longitudinal study demonstrate that baseline RLP cholesterol levels are significantly associated with incident NODAT in RTR, independent of several recognized risk factors. NODAT has substantial clinical relevance predisposing affected RTR to an increased risk of cardiovascular events, graft failure and overall mortality [2, 4]. As a considerable percentage of RTR develop NODAT, it is essential to characterize biomarkers which can identify patients at risk and ideally also serve as target for prevention or therapeutic intervention. Based on our results we believe that RLP cholesterol could be such a biomarker.

RLP cholesterol is an umbrella term for incompletely lipolyzed intestine-derived chylomicrons and liver-derived very low-density lipoproteins. Inadequate lipoprotein lipase activity determined by the expression of the enzyme itself as well as its co-factors is thought to be critical for remnant formation. It was suggested that RLP cholesterol is more damaging than LDL cholesterol due to uncontrolled cellular uptake and therefore it is an increasingly important biomarker in the cardiovascular field [11–13]. However, statins, currently frequently used to treat dyslipidemia, mostly work to decrease LDL cholesterol and have not been unequivocally shown to decrease RLP cholesterol levels [20, 21]. Therefore, as a first step, patients can be advised to implement lifestyle changes such as weight loss, reduction of refined carbohydrate intake and aerobic exercise, and to avoid fructose- and sucrose-sweetened beverages as well as alcohol [22]. In addition, other therapeutic modalities are being developed. In the general population, omega-3 fatty acids are an effective way to lower high RLP cholesterol levels. It was shown that the use of icosapent ethyl significantly reduces the risk of cardiovascular events by effectively decreasing plasma triglyceride concentrations [23]. Although it was not studied whether specifically RLP cholesterol was also reduced, the correlation between triglyceride concentrations and RLP cholesterol makes such an assumption highly plausible [24]. Another potentially useful drug is ezetimibe, which decreases intestinal cholesterol absorption by blocking the Niemann-Pick C1-like-1 cholesterol uptake transporter on enterocytes thereby reducing LDL cholesterol and RLP cholesterol concentrations [25]. Since reducing intestinal cholesterol absorption results in a compensatory increase in cholesterol synthesis, ideally ezetimibe is combined with statins [26], which, however, can increase the risk of NODAT in RTR [8]. Another potential option to reduce circulating RLP cholesterol levels are fibrates. However, their use in RTR has been controversial due to proposed nephrotoxicity and therefore they should be avoided until large randomized controlled trials confirm their safety in the post-transplant population [27]. Novel therapies for treating increased RLP cholesterol concentrations are also emerging. In the general population positive results have been obtained with the use of antisense oligonucleotides targeting hepatic apolipoprotein C-III expression, an endogenous inhibitor of lipoprotein lipase. Studies reported good treatment efficacy as well as increased peripheral insulin sensitivity in patients with type 2 diabetes [28, 29]. Other strategies to increase lipoprotein lipase activity and thereby prevent remnant formation are to lower the expression levels of the inhibitory proteins angiopoietin like 3 and 4 [30]. Although safety has not yet been tested in RTR, no nephrotoxicity was reported in the original studies [31]. Due to the increasing importance of RLP cholesterol, we suggest prospective randomized controlled trials to investigate the efficacy and safety of RLP cholesterol reduction using different therapies in RTR.

TransplantLines is one of the largest longitudinal cohorts of RTR. However, the study is still not sufficiently powered to allow inclusion of a fully adjusted model in Cox regression analyses. Further, TransplantLines is from a single center and the study is observational with the potential for residual confounding. In addition, the study participants largely reflect a White population from the North of the Netherlands. Thus, it would be valuable to validate our results in other populations with different ethnic and socioeconomical backgrounds. Furthermore, RTR were included at least one year after transplantation. Although this might be seen as a limitation of insight into the first year after the transplant procedure, it could also be regarded as a strength since variable transient episodes of hyperglycemia post-transplantation e,g, as a common result of treating phases of acute transplant rejection are not included [32]. In agreement with such reasoning, it has been proposed that NODAT should be diagnosed in stable RTR [32]. Furthermore, please note that in our center prednisolone dosages are on the higher end of the clinical spectrum when compared to other kidney transplant centers. Corticosteroids have a lipid modulating effect and their use can result in higher circulating triglyceride levels potentially indicating elevated RLP cholesterol [22]. However, in our study prednisolone dosages were not different among patients in the different tertiles of RLP cholesterol and the impact of RLP cholesterol on NODAT risk was independent of steroid use. Strengths of our study are the direct measurement of LDL cholesterol, which allows for a precise RLP cholesterol calculation as opposed to the use of e.g. the Friedewald formula [16] as well as the long, thorough and complete follow-up.

## Conclusions

This study demonstrates that baseline plasma RLP cholesterol levels are prospectively associated with incident NODAT in RTR independent of several recognized risk factors such as immunosuppressive medication use. These results position RLP cholesterol as a relatively easy to determine emerging biomarker with an apparent strong clinical impact in RTR. Therefore, we would encourage clinicians to take RLP cholesterol levels into account during routine clinical assessments and to consider lifestyle and pharmacological interventions if levels are increased. More work, however, is required to define a normal range for RLP cholesterol to be handled in clinical settings. Furthermore, close follow-up of RTR with increased RLP cholesterol levels is recommended in order to early identify NODAT with the aim to limit suffering and NODAT-related complications such as graft failure and cardiovascular events.

## Data Availability

The datasets generated and/or analysed during the current study are available from the corresponding author on reasonable request.
